# A Novel Multi-Source Fault Diagnosis Strategy Based on Knowledge and Data Dual-Drive for a Planetary Gearbox

**DOI:** 10.3390/s26102959

**Published:** 2026-05-08

**Authors:** Hanzhi Yang, Jun Hao

**Affiliations:** College of Automation, Jiangsu University of Science and Technology, Zhenjiang 212100, China; 232210305205@stu.just.edu.cn

**Keywords:** knowledge and data dual-drive, multi-source fault diagnosis, multi-source information correlation matrix, planetary gearbox, Softmax logical regression and KNN

## Abstract

Traditional fault diagnosis methods face challenges such as the insufficient utilization of fault information and an imbalance between classification accuracy and computation. To address these issues, a novel multi-source fault diagnosis strategy based on a knowledge and data dual-drive algorithm is proposed. Firstly, a multi-source information correlation matrix (MICM) is designed to enhance the expression of fault information by combining information among time domain, frequency domain and channel correlation features. Then, kernel principal component analysis (KPCA) is used for dimensionality reduction in the MICM. Finally, a novel classifier based on Softmax logical regression (SLR) and K nearest neighbor (KNN) is proposed, where SLR provides an initial pre-classification and KNN is used to achieve accurate classification with less computation. Moreover, the latest planetary gearbox dataset of the wind turbine in a physical experiment is utilized to verify the effectiveness of the proposed MICM-SLR-KNN algorithm, and the experimental results demonstrate the superiority of the algorithm in comparison with other methods.

## 1. Introduction

In recent decades, wind energy as a form of renewable energy has become a cornerstone of global green energy production [1,2,3]. However, the fault downtime of wind turbines caused by the planetary gearbox is increasing, which is directly related to wind energy’s safety and reliability [4]. Hence, it is of great significance to achieve fault diagnosis of wind turbine planetary gearboxes to reduce power loss and ensure equipment safety and wind turbine reliability. Research into fault diagnosis methods is gradually becoming a hot topic for complex systems.

Fault diagnosis methods are usually divided into knowledge-based and data-driven methods for wind turbine planetary gearboxes. In [5], representative knowledge-based KNN is used to classify the main features and confirm the fault classes, and it demonstrates considerable diagnostic efficacy with remarkable precision. An improved KNN algorithm is introduced in [6], which employs cross-validation to determine the optimal value of K and incorporates Gaussian-based distance weighting for neighboring samples. The method in [7] combines multiple KNNs using different distance metrics and adopts a weighted voting scheme based on the individual precision, which enhances predictive precision and robustness. The aforementioned knowledge-based fault diagnosis methods (KFDMs) have achieved good results. However, these approaches usually have a high computational cost to achieve fault diagnosis, which results in inefficiency and a difficulty in adapting to large datasets. Reducing online computation while ensuring precision is an urgent problem that needs to be solved for KFDMs.

Recently, with the development of sensors, fault data has become easier to obtain, making data-driven fault diagnosis methods (DFDMs) attract considerable attention [8,9,10]. Classic data-driven algorithms are often used as benchmarks for building classification models due to their intuitive models and simple implementation. They learn fault patterns from historical data and identify equipment operating conditions. A model of convolutional gated cyclic attention is proposed in [11], with a CNN-BiGRU-CBAM and SLR. A recurrent neural network (RNN) model is constructed for fault diagnosis, which is subsequently enhanced with a fast continuous detection improvement and probability-based correction module [12]. A graph structure is dynamically constructed based on the Transformer multi-head attention mechanism, whose effectiveness is validated using a graph convolutional network (GCN), graph attention network (GAT), and GraphSAGE [13]. Although DFDMs have the advantage of fast online computation, their prediction results have uncertainty, which makes it difficult to ensure the final precision of fault diagnosis. What is more, their performance may tend to degrade when distinguishing similar fault classes [14].

To address the above issues, a novel multi-source fault diagnosis strategy based on knowledge and data dual-drive, namely the MICM-SLR-KNN algorithm, is proposed to achieve intelligent fault diagnosis of the wind turbine planetary gearbox. The proposed algorithm combines the precision of KFDMs and rapidity of DFDMs. Moreover, a novel fault feature extraction matrix, namely MICM, is also designed by combining information among time domain, frequency domain and channel correlation. The main contributions of the paper can be summarized as follows.

(1) Firstly, a novel hybrid classifier, namely SLR-KNN, is proposed, where the aim of SLR is only to predict fault classes with probability distributions while KNN is utilized to achieve precise classification. The proposed SLR-KNN can avoid the online low efficiency of KFDMs while overcoming the diagnostic uncertainty of DFDMs.
(2) In the MICM-SLR-KNN algorithm, a novel fault feature extraction matrix, MICM, is constructed to enhance the expression of fault information, including time domain, frequency domain and channel correlation of vibration and speed signals.
(3) Different from existing standard datasets, the WT-planetary gearbox dataset is collected from the practical experimental platform, including sampling authenticity, data scale, comprehensive coverage of operational frequencies and even interference caused by human assembly.

The article is organized as follows. First, Section 2 introduces the challenges of fault diagnosis for planetary gearbox. Section 3 provides the design of the proposed MICM-SLR-KNN algorithm. Then, Section 4 provides experimental verification and result analysis through the WT-planetary gearbox dataset. Finally, the conclusions are given in Section 5.

## 2. The Challenges of Fault Diagnosis for Planetary Gearbox

**Figure 1:**
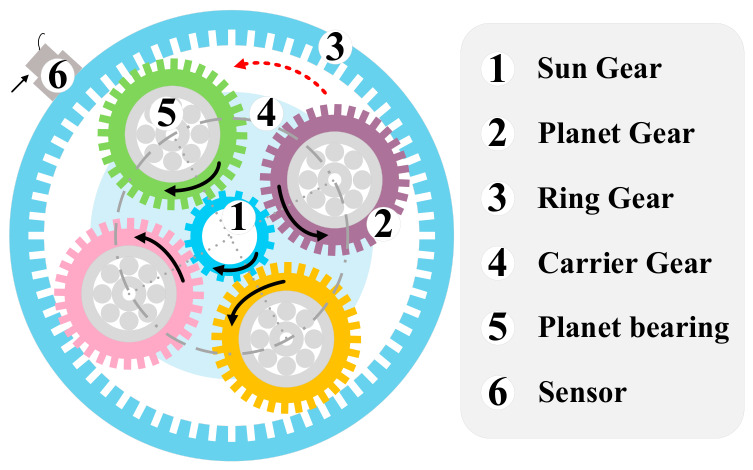
Structure of a planetary gearbox.

The planetary gearbox serves as a critical transmission component in a wind turbine, a exhibits significantly more complex dynamic behavior than the fixed-axis gearbox. As shown in Figure 1, the planetary gearbox consists of a fixed ring gear, a centrally rotating sun gear and multiple planetary gears. Each planetary gear is mounted on a rolling bearing, enabling simultaneous rotation about its own axis and revolution around the sun gear [15]. In such a configuration, the continuous surface contact between gears would give rise to friction, vibration and heat generation, which results in gear wear, breakage and other faults [9]. In addition, these issues can also be accelerated by harsh operating conditions such as high temperature, speed and load [10]. However, due to the complex structure and dynamic features of the planetary gearbox, fault data captured by sensors have the following features [10,16,17] that bring difficulties and challenges to the fault diagnosis of a planetary gearbox.

(1) A planetary gearbox exhibits multiple meshing coupling points with inherent nonlinear signal features. When localized faults occur, the signals manifest a partial imbalance due to the modified dynamic interactions. This is a significant challenge for fault diagnosis to effectively deal with such nonlinear and imbalanced signals.
(2) The complex mechanical structure of the planetary gearbox leads to a multi-path vibration propagation mechanism. The meshing and propagation paths between planetary gears, ring gears and sun gears are complex. There are multiple paths between faults and sensors. This modulation phenomenon may lead to signal aliasing or attenuation, which increases the difficulty of reliable fault diagnosis.
(3) The frequency energy associated with bearing fault features is significantly weaker than that generated by gear meshing phenomena. Such an inherent limitation is further exacerbated by the presence of industrial noise in the practical environment. This would make the identification and extraction of bearing fault features particularly challenging.

Given that gearbox faults may lead to downtime loss, significant maintenance costs and even potential safety incidents, it is essential to establish a rapid and efficient fault diagnosis method for planetary gearboxes to improve the stability and safety of wind turbines.

## 3. Design of the MICM-SLR-KNN Algorithm

**Figure 2:**
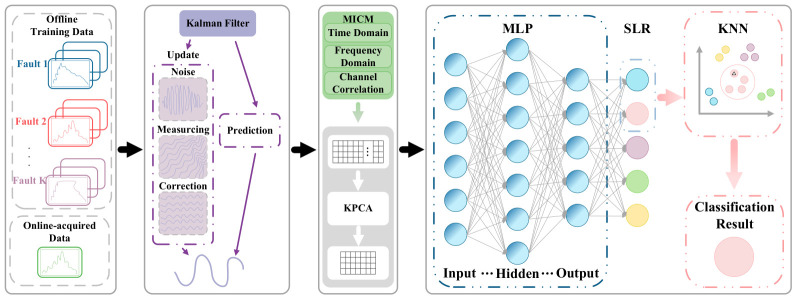
Flowchart of the MICM-SLR-KNN algorithm.

The flowchart of the proposed MICM-SLR-KNN algorithm based on knowledge and data dual-drive is shown in Figure 2. The design process of the MICM-SLR-KNN algorithm mainly consists of two stages, namely offline training and online implementation.

In the offline training stage, the original fault data collected from multiple sensors undergo denoising by Kalman filter and normalization, which reduces the impact of Gaussian noise and dimensional inconsistency. Then, the MICM is established by time domain, frequency domain, and channel correlation analyses on the aforementioned normalized data samples. Afterwards, KPCA is adopted for MICM to reduce redundancy and capture fault features comprehensively. The feature reconstruction loss is defined to determine the output dimension of KPCA. Finally, these low-dimensional features are used to train SLR. After being given by SLR, the class probability would be transferred to KNN to achieve precise fault diagnosis and classification.

In the online implementation stage, the online-acquired data from sensors are processed through a Kalman filter with normalization. Then, the MICM is formed based on normalized data. Then, the low-dimensional features can be obtained via the configured KPCA and fed into SLR-KNN to achieve precise fault diagnosis.

In the MICM-SLR-KNN algorithm, the MICM is established based on knowledge of fault features, while the novel hybrid classifier is designed via knowledge and data dual-drive. The specific descriptions are as follows.

### 3.1. Establishment of the MICM

The original fault data exhibit limited feature diversity, substantial redundant information and Gaussian noise. Consequently, the data are unsuitable to be used directly as fault features in the fault diagnosis. Aimed at comprehensively characterizing the fault information embedded within the fault data, domain knowledge is integrated with feature extraction to construct the MICM, including time domain, frequency domain and channel correlation. Hence, the MICM respectively reveals the variation pattern, energy distribution and information coupling of the fault data.

Time domain features in the MICM reflect the gear meshing dynamics and impact event, which are described as

(1) $$X_{p}=\max(|x_{i}|),$$

(2) $$C_{p}=\frac{X_{p}}{rms},$$

(3) $$C_{pm}=\frac{X_{p}}{\frac{1}{N}\sum_{i=1}^{N}\left|x_{i}\right|},$$

(4) $$C_{ps}=\frac{X_{p}}{{\left(\frac{1}{N}\sum_{i=1}^{N}\sqrt{\left|x_{i}\right|}\right)}^{2}},$$

(5) $$kurtosis=\frac{\frac{1}{N}\sum_{i=1}^{N}{\left(x_{i}-\mu \right)}^{4}}{\sigma^{4}},$$

(6) $$skewness=\frac{\frac{1}{N}\sum_{i=1}^{N}{\left(x_{i}-\mu \right)}^{3}}{\sigma^{3}},$$

where $x_{i}$ is the channel fault data for feature extraction with root mean square rms, mean μ, and standard deviation value σ, respectively. The peak value $X_{p}$ captures the maximum impact amplitude. The crest factor $C_{p}$ reflects the severity of impulsive components. The impulse factor $C_{pm}$ can effectively detect sudden impulses even under very small impact conditions. The margin factor $C_{ps}$ reflects how prominently the impulsive peaks stand out relative to the overall vibration level. The kurtosis calculates the presence of heavy-tailed and impact-dominated vibration. The skewness measures the asymmetry of the waveform.

The frequency domain features incorporate mechanical knowledge of meshing dynamics, where the energy distribution reveals fault features. The selected segment of the sensor fault data is transformed as the input data using FFT to obtain its frequency spectrum $X(f_{k})$. The corresponding frequency sequence $f_{k}$ is defined as

(7) $$f_{k}=\frac{kf_{s}}{N},k=0,1,\ldots ,\frac{N}{2}-1,$$

where $f_{s}$ is the sampling frequency and N is the data length.

The power spectral density (PSD) is used to describe the power distribution and is calculated as

(8) $$P(f_{k})=\frac{|X(f_{k}){|}^{2}}{N}.$$

The frequency features considered in the paper are presented as

(9) $$FC=\frac{\sum_{k=0}^{N/2-1}f_{k}P(f_{k})}{\sum_{k=0}^{N/2-1}P(f_{k})},$$

(10) $$MSF=\frac{\sum_{K=0}^{N/2-1}f_{k}^{2}P(f_{k})}{\sum_{K=0}^{N/2-1}P(f_{k})},$$

(11) $$VF=\frac{\sum_{k=0}^{N/2-1}{\left(f_{k}-FC\right)}^{2}P(f_{k})}{\sum_{k=0}^{N/2-1}P(f_{k})},$$

where the frequency center FC describes the weighted average frequency of the spectrum. The mean square frequency MSF measures the concentration of energy in higher frequency bands. The frequency variance VF quantifies how widely the spectral energy is distributed around the average frequency.

During the operation of the wind turbine planetary gearbox, periodic impact vibration signals are generated when the fault occurs. They contain distinct pulse structures and characteristic frequencies in the vibration waveforms. However, due to the complex operating environment and strong Gaussian noise [18], the periodic features of the original fault data are often difficult to identify directly. The autocorrelation can capture periodic features and effectively suppress Gaussian noise [19], which can be calculated as

(12) $$R_{xx}(\tau )=\sum_{t}\left(x(t)-\bar{x}\right)(x(t+\tau )-\bar{x}).$$

To leverage the property, the autocorrelation peak is adopted as a fault feature and calculated as

(13) $$P_{auto}=\max(R_{xx}(\tau )),\tau >0.$$

The multi-channel fault data are inherently coupled [20]; therefore, the cross-correlation peak is selected as a key feature to exploit similarity between data [21], which can be obtained as

(14) $$R_{xy}(\tau )=\sum_{t}\left(x(t)-\bar{x}\right)(y(t+\tau )-\bar{y}),$$

(15) $$P_{cross}=\max(R_{xy}(t)),$$

where x, y are fault data from different sensor channels.

In summary, the MICM contains rich fault features and provides a solid foundation for subsequent dimensionality reduction and classification.

### 3.2. Design of Hybrid Classifier Based on Knowledge and Data Dual-Drive

When the low-dimensional features are obtained after KPCA, reliable and effective classifiers become crucial. A novel hybrid classifier SLR-KNN is proposed here, where SLR is trained and established using offline training to achieve pre-classification with probability distributions, while KNN achieves precise classification through similarity calculation between samples and nearest neighbor discrimination rules.

#### 3.2.1. Pre-Classification of SLR

As the initial stage of the hybrid classifier, SLR partitions all input samples into probabilistically assigned subsets. The process establishes refined sample domains and reduces the search space for precise classification by KNN.

The SLR is implemented using a multi-layer perceptron (MLP). Given the low-dimensional feature vector $m\in R^{d}$, the hidden layer applies a nonlinear transformation

(16) $$h=\phi (W_{1}m+b_{1}),$$

where ϕ(⋅) denotes an element-wise nonlinear mapping. h is the feature representation of the hidden layer. W and b are weight and bias, respectively. $W_{1}$ and $b_{1}$ are initialized by Kaiming. ϕ(⋅) employs RELU here.

The output layer then calculates the class logits

(17) $$o=W_{2}h+b_{2},$$

where $W_{2}$ and $b_{2}$ are initialized by Xavier.

Let us substitute (17) into (18):

(18) $$q(o_{i})=\frac{e^{o_{i}}}{\sum_{i=1}^{C}e^{o_{i}}},$$

where q(⋅) transforms outputs of the output layer into probabilities between 0 and 1. C is the class number.

To measure the discrepancy between the predicted class and the truth class, the cross-entropy loss is adopted as the training objective

(19) $$L(\Theta )=-\frac{1}{M}\sum_{i=0}^{M-1}\sum_{k=0}^{C-1}\varphi_{k}\log q(o_{k}),$$

where M is the number of fault samples. $\varphi_{k}=1$ when k is the true class and $\varphi_{k}=0$ otherwise.

During training, the algorithm parameters are iteratively updated using the AdamW optimizer to minimize (19). For each batch, parameter gradients are obtained via back-propagation, and the update rule is expressed as

(20) $$\Theta^{\left(t+1\right)}=\Theta^{\left(t\right)}-\eta {\hat{g}}^{\left(t\right)},$$

where η is the learning rate and ${\hat{g}}^{\left(t\right)}$ denotes the bias-corrected gradient estimate provided by the optimizer.

The trained SLR outputs a probability vector for each

(21) $$\hat{n}=\arg\;\max(q),$$

where $\hat{n}$ is the class corresponding to the higher probability.

For each predicted class, the corresponding training subset

(22) $$D_{k}=\{(m,z)|\hat{n}=k\}$$

is constructed to provide a more localized search space for KNN, where z is the true class of m.

A separate KNN is constructed for each $D_{k}$ to store and manages its corresponding feature vectors.

#### 3.2.2. Precise Classification by KNN

During online implementation for fault diagnosis, an online-acquired sample $m^{*}$ is sent to the hybrid classifier after data preprocessing and feature extraction. It first passes through the trained SLR to obtain ${\hat{n}}_{k}$ with a higher possibility. The sample is then sent into KNN and the Euclidean distance is calculated by:

(23) $$d(m^{*},m)=||m^{*}-m|{|}_{2},m\in D_{k}.$$

KNN prediction is achieved by:

(24) $$\hat{z}=\arg\;\underset{k}{\max}\sum_{m\in N(m)}1(z=k)$$

where N denotes the set of the K nearest neighbors of $m^{*}$. 1(⋅) is the indicator function returning 1 if true, and 0 otherwise.

If $\hat{z}={\hat{n}}_{k}$, the final decision is $\hat{z}$. Otherwise, ${\hat{n}}_{k^{\prime }}$ corresponding to the second higher probability of SLR is obtained. The above steps are repeated to get a new prediction ${\hat{z}}^{\prime }$. If ${\hat{z}}^{\prime }={\hat{n}}_{k^{\prime }}$, ${\hat{z}}^{\prime }$ is taken as the final output; otherwise, $m^{*}$ is defined as an outlier.

The pseudocode for the MICM-SLR-KNN algorithm [Algorithm 1] is as follows.
**Algorithm 1** Outline of SLR-KNN hybrid classifier for planetary gearbox fault diagnosis**Offline** SLR training and separate KNN construction based on class probabilities **1.**      The input training fault samples [m,z], class number C, epoch number E and learning rate η are obtained. **2.**                  construct Dataloader for training fault samples**3.**                  initialize MLP network using (16), (17)**4.**                  initialize AdamW with η**5.**                  bestP←0**6.**                  **for** epoch=1 to E**7.**                         q is calculated by (18)**8.**                         q is fed into (19)**9.**                         update parameters using (20)**10.**                       $\hat{n}$ is obtained by (21)**11.**                       compute validation precision P**12.**                       **if** P>bestP**13.**                              bestP←P**14.**                              **for** each training sample**15.**                                    save m, $\hat{n}$ and z**16.**                              **end****17.**                              **for** k=0 to C−1**18.**                                    **if** $\hat{n}==k$**19.**                                          store [m,z] into $D_{k}$**20.**                                    **endif****21.**                              **end****22.**                       **endif****23.**                **end****24.**                construct separate KNN for $D_{k}$**25.**    The trained SLR and the separate KNN are the outputs.**Online** Real-time classification of online-acquired fault sample using SLR-KNN hybrid classifier
**26.**    The online prediction receives an online-acquired fault sample $\left[m^{*},z\right]$. The trained SLR and separate KNN for $D_{k}$ are also obtained. **27.**                $m^{*}$ undergoes the data preprocessing and feature extraction**28.**                $\hat{n}$ is obtained by (16), (17), (18), (21)**29.**                $m^{*}$ and $m\in D_{k}$ are fed into (23)**30.**                $\hat{z}$ is obtained by (24)**31.**                **if** $\hat{z}=={\hat{n}}_{k}$**32.**                       the final prediction is $\hat{z}$**33.**                **elseif****34.**                       ${\hat{n}}^{\prime }$ is obtained**35.**                       do steps 29 to 30 again and ${\hat{z}}^{\prime }$ is obtained**36.**                       **if** ${\hat{z}}^{\prime }=={\hat{n}}_{k^{\prime }}$**37.**                              the final prediction is ${\hat{z}}^{\prime }$**38.**                       **elseif****39.**                              $m^{*}$ is defined as an outlier**40.**                       **endif****41.**                **endif****42.**    The final classification result of $m^{*}$ is output.

## 4. Experimental Verification and Result Analysis

Different from existing standard fault datasets, many practical factors are accounted for in the WT-planetary gearbox dataset [22], such as sampling authenticity, data scale, comprehensive coverage of operational frequencies and interference caused by human assembly. Hence, such a WT-planetary gearbox dataset is used as the data source of the MICM-SLR-KNN algorithm, which can fully verify the reliability and effectiveness of the proposed algorithm in the practical experimental platform. It is openly available in GitHub at https://github.com/Liudd-BJUT/WT-planetary-gearbox-dataset (accessed on 23 July 2025) [22].

**Figure 3:**
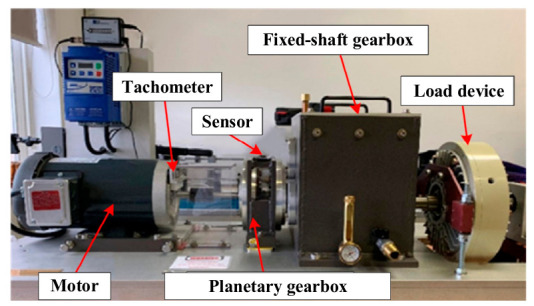
Experimental platform.

**Figure 4:**
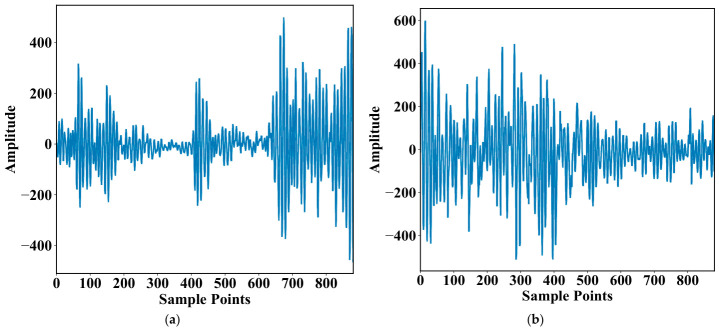
Representative vibration signals of different faults: (**a**) vibration signals of fault B; (**b**) vibration signals of fault W.

The WT-planetary gearbox platform is shown in Figure 3 [22]. Five typical pieces of fault information are obtained by sensors, including healthy state (N), broken tooth (B), worn tooth (W), root crack (R), and missing tooth (M). The multi-source information is recorded, including vibration signals and speed pulse signals. The gearbox operates at eight rotational speeds ranging from 20 to 55 Hz under two different load conditions, so as to construct experimental conditions with rich operating condition coverage. Figure 4 shows one period of vibration signals for fault B and W at a rotational speed of 55 under the same load condition. Moreover, the sampling time of each operating condition is more than five minutes. Not only does this ensure sufficient signal size, it also preserves the integrity of the signal in the construction of the MICM.

Two typical algorithms, namely PSAL-SLR [23] and DTW-KNN [24], are used for comparison to demonstrate the superiority of the proposed MICM-SLR-KNN algorithm. The precision P and recall R are adopted as criteria, and their expressions are defined as

(25) $$P=\frac{TP}{TP+FP}\times 100\%,$$

(26) $$R=\frac{TP}{TP+FN}\times 100\%,$$

where TP, FP, and FN represent true positive, false positive and false negative, respectively. Their standard deviations, $\sigma_{P}$ and $\sigma_{R}$, are also used as criteria to assess the stability of the method. The expression is expressed as

(27) $$\sigma =\sqrt{\frac{1}{K-1}\sum_{i=1}^{K}{\left(a_{i}-\bar{a}\right)}^{2}},$$

where $a_{i}$ is the P or R for each experimental operation. K represents the number of experimental operations.

To accurately analyze the contribution of features from different domains to the P and R of the MICM-SLR-KNN algorithm, ablation experiments are also conducted using only single domain features in the paper.

### 4.1. Experimental Results of a Single-Operation Condition

**Figure 5:**
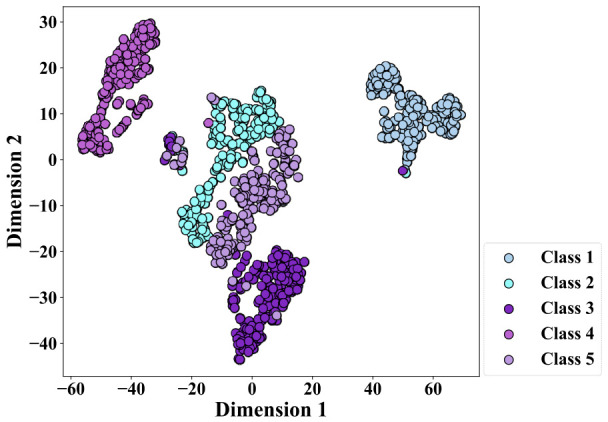
T-SNE visualization of low-dimensional features under the single-operation condition.

**Figure 6:**
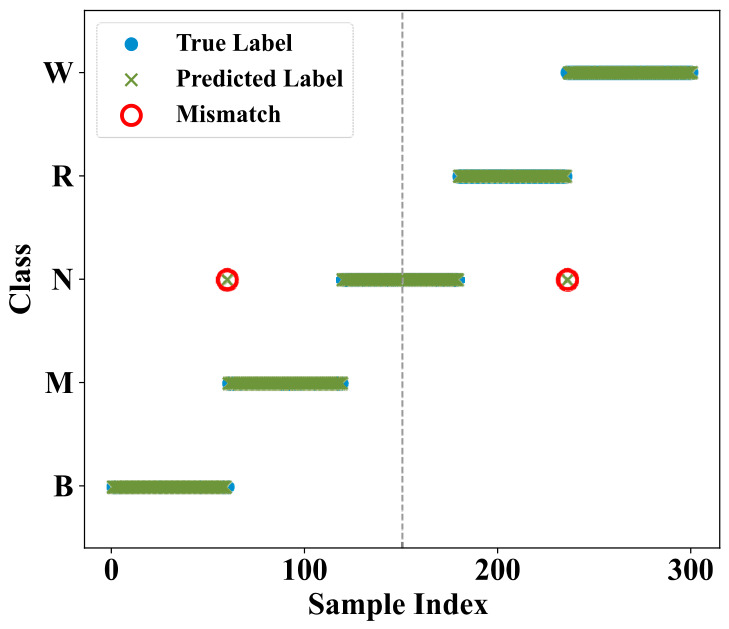
True and predicted labels for the online-acquired data ordered by class under the single-operation condition.

**Figure 7:**
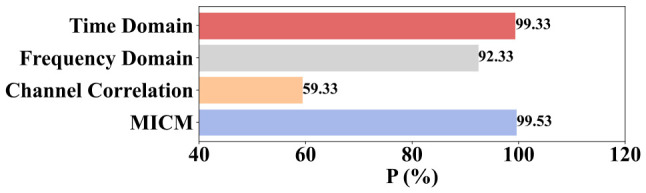
Ablation experiment of different domain features under the single-operation condition.

Firstly, the experimental results are validated on a single-operation condition, where the rotational speed is 55 Hz. In this case, the process noise and measurement noise are set to 0.1 and 5, based on empirical observations, to balance noise suppression and fault information fidelity. The output dimension of KPCA is determined based on the feature reconstruction loss,

(28) $$\varepsilon =1-\frac{\sum_{i=1}^{s}\lambda_{i}}{\sum_{i=1}^{S}\lambda_{i}},$$

where λ is the eigenvalue. s is the output dimension of KPCA, and S is the original dimension of the MICM. It is set to 20, due to the fact that ε is limited within 0.03. The parameters of neural networks are selected through a trial and error method, such as the size of hidden layer and the learning rate. The number of neurons in the hidden layer is 16 and the learning rate is 0.1. A similar case is adopted for K in KNN, and the K in KNN is 1. In the PSAL-SLR algorithm, the size of the hidden layer and the learning rate are set to 128 and 0.00004, respectively. K in the DTW-KNN algorithm is set to 3. The experimental results of the MICM-SLR-KNN algorithm are shown in Figure 5 and Figure 6. Experimental results of different algorithms are listed in Table 1. The relevant results of the ablation experiment are shown in Figure 7.

The t-SNE visualization of the features extracted by MICM-KPCA is shown in Figure 5.

From Figure 5, one can see that the feature space exhibits a clear separation among the five fault types.

The classification result of the MICM-SLR-KNN algorithm on the online-acquired data is shown in Figure 6.

From Figure 6, the MICM-SLR-KNN algorithm has good fault diagnosis ability. Most fault samples are predicted accurately.

To fully demonstrate the effectiveness of the MICM-SLR-KNN algorithm and avoid randomness of the experimental results, the average P and R of the fifteen experimental operations serve as the final criteria and are listed in Table 1. The standard deviations $\sigma_{P}$ and $\sigma_{R}$ are also listed. T represents the time of a single period.

From Table 1, the proposed MICM-SLR-KNN algorithm demonstrates superior performance to the other two algorithms in terms of both computation and diagnostic effectiveness. Furthermore, the standard deviations of the precision and recall are relatively low, indicating the stability of the proposed algorithm.

The P of the MICM-SLR-KNN algorithm based on different single domain features and the MICM is shown in Figure 7, whose condition is single-operation.

From Figure 7, one can see that time domain and frequency domain features contribute significantly to the experimental results. Channel correlation features contribute moderately.

### 4.2. Experimental Results of Multiple-Operation Condition

Due to the multiple operating frequencies and complex fault information, the fault diagnosis of the multiple-operation condition is more challenging. The rotational speeds are from 20 to 55 Hz. In this case, according to the method of setting parameters under the single-operation condition, the process noise and measurement noise are 0.1 and 50, respectively. The output dimension of KPCA is 70. The number of neurons in the hidden layer is 200 and the learning rate is 0.05. K is set to 1. The size of the hidden layer and the learning rate in the PSAL-SLR algorithm are set to 128 and 0.00004, respectively. K in the DTW-KNN algorithm is set to 3.

**Figure 8:**
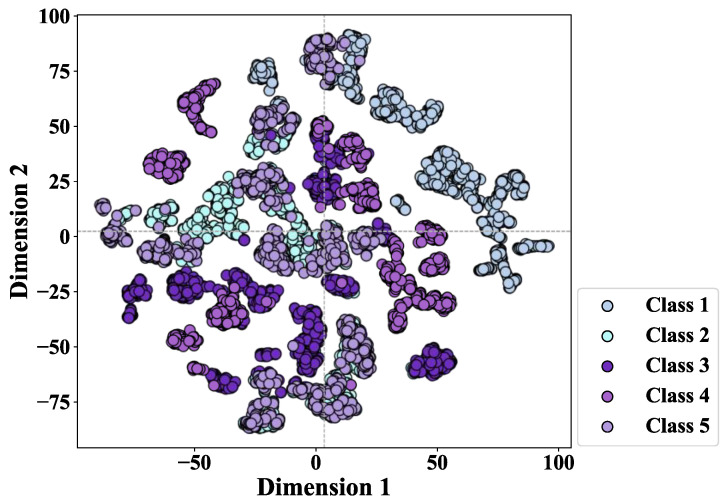
T-SNE visualization of low-dimensional features under multiple-operation conditions.

**Figure 9:**
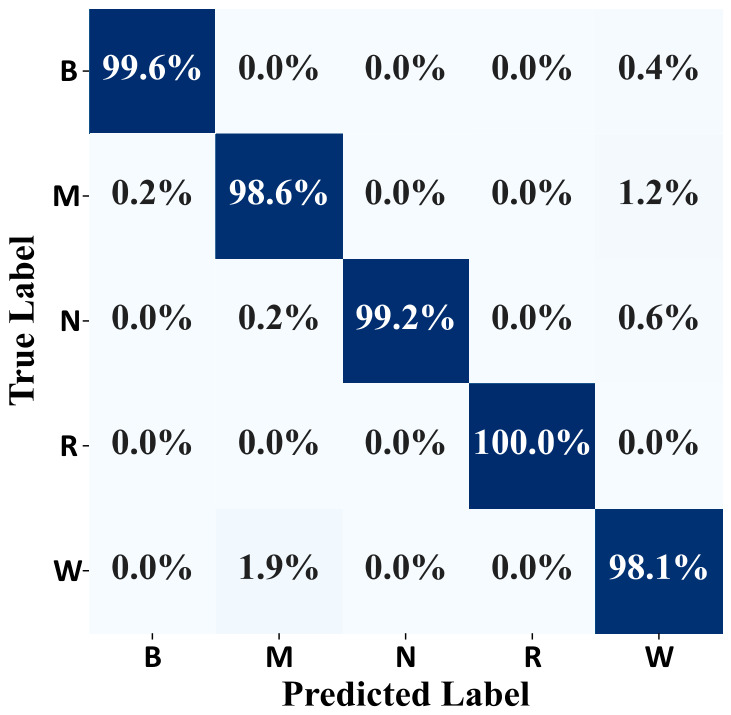
Confusion matrix for the MICM-SLR-KNN algorithm under the multiple-operation condition.

**Figure 10:**
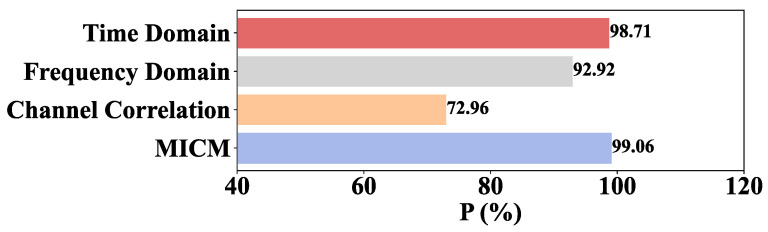
Ablation experiment of different domain features under multiple-operation conditions.

The relevant experimental results are presented in Figure 8 and Figure 9. The comparison results of different algorithms are listed in Table 2. The relevant results of the ablation experiment are shown in Figure 10.

The t-SNE visualization of the low-dimensional features is shown in Figure 8.

From Figure 8, it can be seen that the MICM-KPCA can still effectively group fault samples from identical faults within similar operating conditions, revealing its sensitivity to the condition and consistency of clustering.

The classification performance of the MICM-SLR-KNN algorithm is shown in Figure 9.

From Figure 9, the MICM-SLR-KNN algorithm has a good classification performance for each class of fault. The average results and standard deviations of fifteen experimental operations for three algorithms are listed in Table 2. The time to make an online prediction is also listed.

From Table 2, the MICM-SLR-KNN algorithm demonstrates superior fault diagnosis performance, with notably higher precision and recall compared to the PSAL-SLR and DTW-KNN algorithm. Additionally, it has significant advantages in online computation speed, which ensures online applicability performance. The low standard deviation indicates that the algorithm is stable, avoiding the randomness of the results.

Figure 10 shows the P of the MICM-SLR-KNN algorithm using single domain features under multiple-operation conditions.

From Figure 10, the time domain and frequency domain features can significantly impact the experimental results, while channel correlation features have only a moderate effect.

### 4.3. Result Analysis

The time domain, frequency domain and channel correlation features are extracted from multi-source vibration and speed signals to construct the MICM. It comprehensively expresses multi-source information and allows for the effective separation of fault samples from different fault classes within the low-dimensional feature space of KPCA. The t-SNE visualizations of features extracted by MICM-KPCA are also shown in Figure 5 and Figure 8.

In terms of the classifier, a novel SLR-KNN hybrid classifier is proposed to solve the uncertainty issue of SLR and the low efficiency of KNN. In the SLR-KNN, SLR is used to obtain fault classification with probability distributions, which provides the criterion of classification priority for KNN to reduce the time consumption of traversal searching. Meanwhile, KNN, as an effective classification algorithm, can be regarded as double-checked validation of SLR, which solves the uncertainty of SLR classification results. The results of fault diagnosis for proposed MICM-SLR-KNN algorithm can be seen in Figure 6, Figure 7, Figure 9 and Figure 10, and Table 1 and Table 2.

The online computation time of the MICM-SLR-KNN algorithm is slightly increased in comparison with existing DFDMs. However, it would not affect the online applicability of the MICM-SLR-KNN algorithm, and the related results as shown in Table 1 and Table 2.

In the experimental simulation section, a typical dataset from experiments under single-operation conditions and multiple-operation conditions, from the practical WT-planetary gearbox shown in Figure 3, is assembled to further validate the superiority of the MICM-SLR-KNN algorithm. Such a dataset contains a rich variety of fault types, all of which are practically and directly collected by sensors. Hence, the practical WT-planetary gearbox dataset is also crucial for verifying the authenticity and effectiveness of the algorithms. Compared to the PSAL-SLR and DTW-KNN algorithms, the proposed MICM-SLR-KNN algorithm achieves better results, which further proves the superiority of the MICM-SLR-KNN algorithm in practical engineering.

## 5. Conclusions

In this manuscript, a novel MICM-SLR-KNN algorithm based on knowledge and data dual-drive is proposed to achieve the precise fault diagnosis of a practical WT-planetary gearbox. Unlike existing KFDMs and DFDMs, SLR of the proposed algorithm only lists possible classifications, while KNN is used to achieve precise classification by verifying two classes with the higher possibility. The proposed algorithm effectively mitigates the uncertainty of SLR accuracy while overcoming the inefficiency of KNN. Moreover, the fault information enhancement matrix, MICM, is designed to further improve the extraction of optimal fault features. Experimental simulations have verified the superiority and online applicability of the MICM-SLR-KNN algorithm. In the future, the MICM-SLR-KNN algorithm would be validated on larger and more extensive datasets and developed for practical systems.

## Figures and Tables

**Table 1:** Comparison of different algorithms under the single-operation condition.

	MICM-SLR-KNN	PSAL-SLR [23]	DTW-KNN [24]
T	0.12 s	0.11 s	23.00 s
P	99.53%	90.92%	92.00%
$\sigma_{P}$	0.17%	4.18%	-- ^1^
R	99.53%	91.45%	92.73%
$\sigma_{R}$	0.17%	4.40%	-- ^1^

^1^ DTW-KNN is a deterministic algorithm. When run multiple times with the same fault data, P and R are exactly the same. Its standard deviation is undefined and is 0.

**Table 2:** Comparison of different algorithms under multiple-operation conditions.

	MICM-SLR-KNN	PSAL-SLR [23]	DTW-KNN [24]
T	0.38 s	0.31 s	184.24 s
P	99.06%	88.39%	81.04%
$\sigma_{P}$	0.17%	4.41%	--
R	99. 06%	88.88%	81.53%
$\sigma_{R}$	0.17%	4.43%	--

## Data Availability

The original data presented in the study are openly available in GitHub at https://github.com/Liudd-BJUT/WT-planetary-gearbox-dataset (accessed on 23 July 2025).

## Abbreviations

The following abbreviations are used in this manuscript:

MICM	Multi-source information correlation matrix
KPCA	Kernel principal component analysis
SLR	Softmax logical regression
KNN	K nearest neighbor
KFDM	Knowledge-based fault diagnosis method
DFDM	Data-driven fault diagnosis method
RNN	Recurrent neural network
GCN	Graph convolutional network
GAT	Graph attention network
PSD	Power spectral density
MLP	Multi-layer perceptron
N	Healthy state
B	Broken tooth
W	Worn tooth
R	Root crack
M	Missing tooth
